# A Brief Review of Critical Processes in Exercise-Induced Muscular Hypertrophy

**DOI:** 10.1007/s40279-014-0152-3

**Published:** 2014-05-03

**Authors:** Stuart M. Phillips

**Affiliations:** Exercise Metabolism Research Group, Department of Kinesiology, McMaster University, 1280 Main Street West, Hamilton, ON L8S 4K1 Canada

## Abstract

With regular practice, resistance exercise can lead to gains in skeletal muscle mass by means of hypertrophy. The process of skeletal muscle fiber hypertrophy comes about as a result of the confluence of positive muscle protein balance and satellite cell addition to muscle fibers. Positive muscle protein balance is achieved when the rate of new muscle protein synthesis (MPS) exceeds that of muscle protein breakdown (MPB). While resistance exercise and postprandial hyperaminoacidemia both stimulate MPS, it is through the synergistic effects of these two stimuli that a net gain in muscle proteins occurs and muscle fiber hypertrophy takes place. Current evidence favors the post-exercise period as a time when rapid hyperaminoacidemia promotes a marked rise in the rate of MPS. Dietary proteins with a full complement of essential amino acids and high leucine contents that are rapidly digested are more likely to be efficacious in this regard. Various other compounds have been added to complete proteins, including carbohydrate, arginine and glutamine, in an attempt to augment the effectiveness of the protein in stimulating MPS (or suppressing MPB), but none has proved particularly effective. Evidence points to a higher protein intake in combination with resistance exercise as being efficacious in allowing preservation, and on occasion increases, in skeletal muscle mass with dietary energy restriction aimed at the promotion of weight loss. The goal of this review is to examine practices of protein ingestion in combination with resistance exercise that have some evidence for efficacy and to highlight future areas for investigation.

## Introduction

The process of skeletal muscle protein turnover is constant and ongoing. Protein turnover within muscle is the sum of the processes of both muscle protein synthesis (MPS) and muscle protein breakdown (MPB). Beyond childhood growth, chronic imbalances between the processes of MPS and MPB lead to a net gain in protein pool size (hypertrophy: MPS > MPB) or a net loss (atrophy: MPB > MPS). Often, athletes seek to maximize a hypertrophic response to exercise with the general acceptance that this may translate into performance gains. Hypertrophy, or the offsetting of atrophy, may also be a goal for athletes in recovery from injury, and so understanding the mechanisms that regulate muscle mass are important. The goal of this review is to provide a brief overview of the factors that regulate hypertrophy and how they can be affected by nutritional factors with a focus on protein.

## Regulation of Muscle Protein Turnover

Resistance exercise provides a loading stimulus to skeletal muscle that results in increases in skeletal MPS and, if performed in the fasted state, an increase in MPB [1, 2]. The increase in fasted-stated MPS with resistance exercise is long-lasting and persists for at least 48 h [1], and maybe longer with a higher volume of focused contractions [3]. Provision of amino acids intravenously [4, 5], as isolated proteins [6–8], or in foods such as beef [9] and milk [10] that promote hyperaminoacidemia and hyperinsulinemia are all effective in stimulating MPS. In addition, post-exercise hyperaminoacidemia suppresses the rise in MPB [4] that occurs following resistance exercise in the fasted state [1, 2]. Post-exercise hyperinsulinemia is not overtly stimulatory for MPS [11], but will also simultaneously suppress MPB [11]. It thus appears that rather than being strictly anabolic, the hyperinsulinemia that accompanies post-exercise protein consumption is not stimulatory but probably merely permissive for MPS [12] and suppressive for MPB [13, 14]. Therefore, when protein is ingested after resistance exercise it is the amino acids themselves that are driving the rise in post-exercise MPS [4, 15]. It is also now quite clear that it is really only the essential amino acids (EAA) that drive the process of MPS [16, 17]. However, perhaps more important is that the key EAA is leucine, as it alone appears to be the metabolic trigger for MPS [18, 19].

**Fig. 1:**
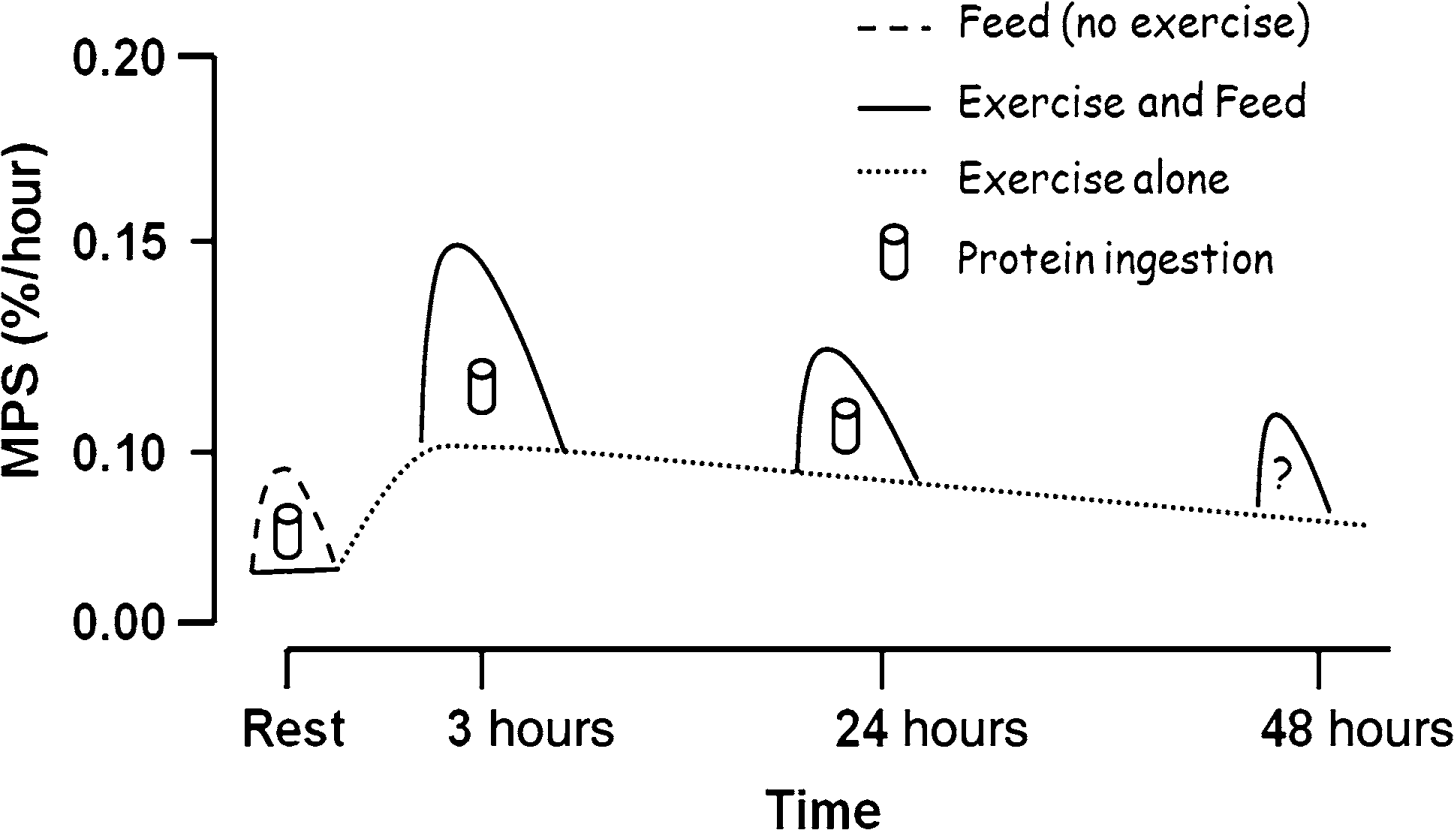
Resistance exercise stimulates a prolonged elevation of MPS that can remain elevated for at least 48 h (*dotted line*) [1]. Protein ingestion at any point during this enhanced period of ‘anabolic potential’ will have an additive effect to these already elevated exercise mediated rates (*solid lines*) [26, 27]. Reproduced from Churchward-Venné et al. [29], with permission. *MPS* muscle protein synthesis

A complete mechanistic explanation of muscle protein turnover and its regulation is beyond the scope of this review; however, several reviews have covered this topic in detail and provide an excellent background [20, 21]. With feeding, we now know that meal-to-meal fluctuations in MPS dictate the fed-state gains, and fasted-state losses, in muscle protein [22–24]. Resistance exercise amplifies the inherent feeding response, which is actually quite transient [25], both immediately after exercise [25, 26] and at 24 h post-exercise [27]. An important study in this area was performed by Tipton et al. [28], who showed that 24-h net protein balance reflected the acute changes in muscle protein turnover induced by both aminoacidemia and resistance exercise. However, Fig. 1 highlights the fact that the MPS response to aminoacidemia post-exercise wanes with time and the acute period post-exercise appears to be an optimal time to ingest protein-promoting hyperaminoacidemia and a robust stimulation of MPS [29]. As mentioned, the nascent stimulation of MPS from resistance exercise alone lasts at least 24 h [1]. We have thus recently proposed that an enhanced amino acid sensitivity of protein synthesis in this window of ‘anabolic potential’ probably persists for just as long (Fig. 1). However, the mechanisms for enhanced sensitivity to amino acid feeding at each timepoint may be different, with the intriguing hypothesis that at later times (i.e. 24 h and beyond) following resistance exercise amino acid transport may be enhanced [30, 31].

## Dose–Response of Dietary Protein and MPS

To date only three true dose–response studies in which MPS has been measured have been published [15, 32, 33]. In those studies, the main message was that MPS is a saturable process in young people at protein ingestion doses of approximately 20–25 g (~8.5–10 g of EAA) regardless of whether the subjects exercised [15] or not [32]. Moore et al. [15] also noted that, in parallel with the rise in MPS, the albumin protein synthetic rate showed a strikingly similar saturable dose–response curve, demonstrating that at least one other body protein had similar synthetic kinetics. In an attempt to standardize this protein dose to body mass (BM), and using the subjects’ mass from the study by Moore et al. [15], the dose of protein that was maximally effective (20 g) post-exercise equated to approximately 0.25 g protein/kg BM. While egg was the protein source used in that study [15], the rationale being that it is the internationally recognized standard protein, similar data would be expected with other high-quality proteins. However, the dose of protein that is maximally stimulatory in older adults is closer to 40 g following resistance exercise and 20 g at rest [33]. Beyond the levels at which MPS is maximally stimulated, it has been noted that the oxidation of an indicator amino acid, leucine, rises quite sharply [15, 33], indicating that amino acids are not being used for protein synthesis and instead are oxidized, probably leading to urea production. While oxidative amino acid loss has been used as an indication of protein excess, it may well be that oxidative losses would still occur despite the fact that protein synthesis is not maximally stimulated as a result of lower *K*_m_ (Michaelis–Menten kinetics—the substrate concentration at which the reaction rate is half of maximal) values of enzymes involved in amino acid degradation compared with, for example, the *K*_m_ for the activation of mammalian target of rapamycin [34, 35]. The traditional interpretation of amino acid oxidation as being ‘wasteful’ may not be a true sentiment where optimal stimulation of MPS is concerned.

## Protein Quality and Muscle Protein Turnover

Protein quality has traditionally been defined by the protein digestibility-corrected amino acid score (PDCAAS). This estimate of quality is derived from measures of the limiting EAA content in the protein compared with that of a reference protein (egg protein) multiplied by the digestibility of the protein [36]. However, issues with the PDCAAS method of scoring proteins have been raised and relate to the validity of the preschool-age child amino acid requirement values, the use of fecal versus ileal digestibility and the truncation of values at 1.0 (i.e. the EAA content of proteins greater than that of egg protein are not important for tissue growth or maintenance) [36, 37]. The restriction of a PDCAAS value at 1.0 obscures the fact that the content of particular amino acids, such as leucine, are higher in the milk-derived proteins casein and whey compared with that of soy by 33 % and 76 %, respectively [38]. This difference in leucine content probably has some functional significance because leucine has been shown to be an important regulatory activator of skeletal MPS [18, 19, 39, 40]. It has recently been reported that even small doses of protein, that were only 25 % of the maximally effective protein dose for stimulating MPS [15], could be made to be maximally effective with the addition of leucine [41]. Therefore, despite an equivalent PDCAAS score it is perhaps not surprising that whey was found to be superior to soy protein in stimulating MPS in both a rested and contracted muscle [6]. Interestingly, the same result was found in older men [42].

While isolated proteins are an interesting model, most athletes consume whole foods. It was previously shown that skimmed milk was superior to a nutrient-matched soy beverage [10], which was also attributed to the high leucine content of milk proteins in the 4:1 ratio of casein:whey in bovine milk. Of note, whey was also found to be superior to casein in stimulating MPS in both rested and contracted muscles [6]. This is an interesting observation given that the leucine content of whey is only 20 % higher than that of casein. However, casein is digested much more slowly than whey and has even been termed a ‘slow’ protein by comparison to whey, which is an acid-soluble and rapidly-digested protein [43]. Similar to the finding of the author’s group [6], Pennings et al. [7] recently reported that whey was superior to both casein and casein hydrolysate in stimulating muscle protein accretion. Therefore, even hydrolysis (i.e. pre-digestion) of casein to speed up its digestion did not result in a greater stimulation of MPS [7]. When protein was fed in small pulses, resulting in protracted hyperaminoacidemia with low amplitude, compared with a large bolus, with rapid and transient aminoacidemia with larger amplitude, a smaller rise in MPS occurred [44].

**Fig. 2:**
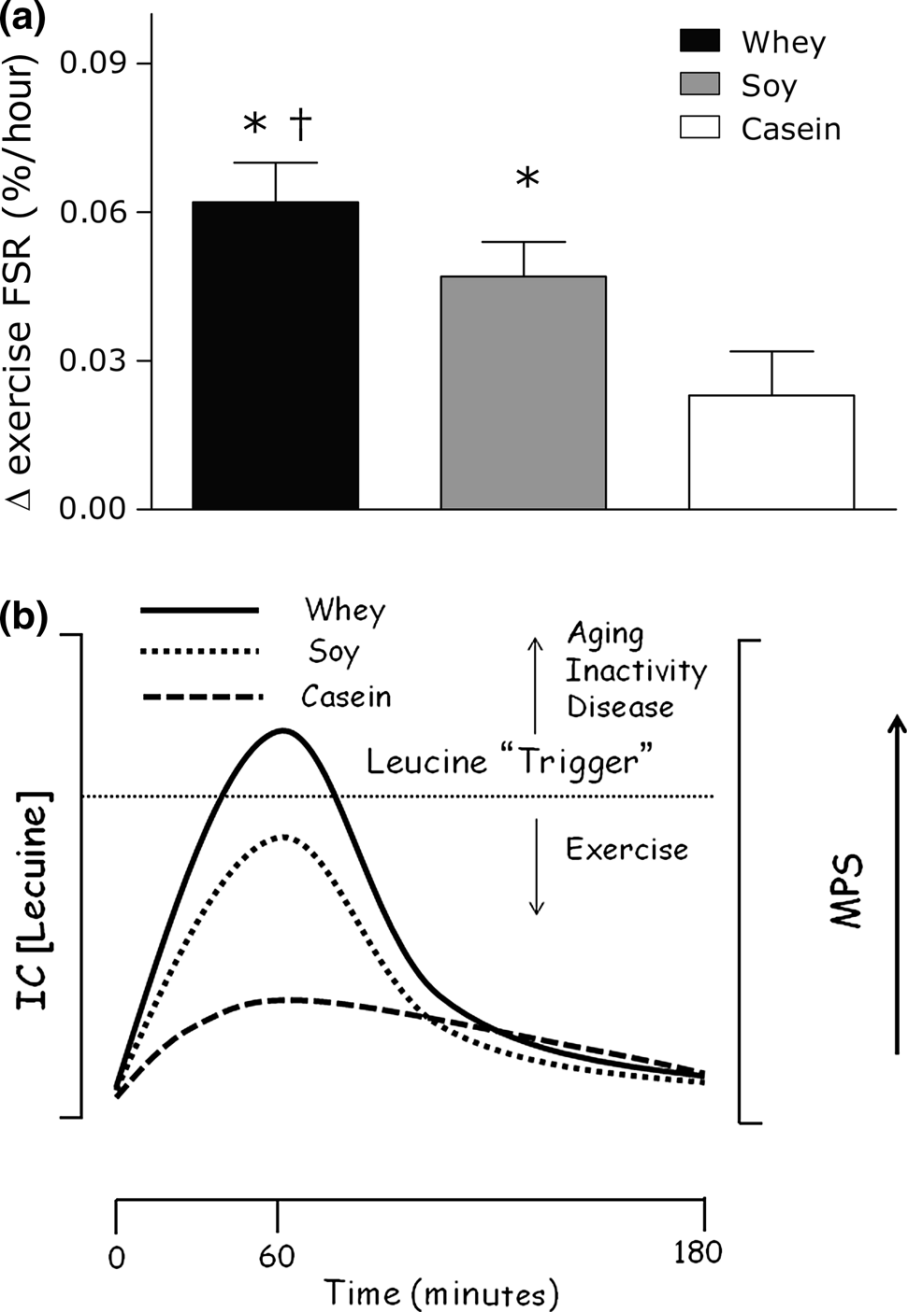
**a** The ‘leucine trigger’ concept, with data adapted from Tang et al. [6], as shown for isolated whey protein, soy protein, and casein proteins as a difference between rested and exercise values for MPS. **b** The speed of digestion of these proteins would be digested in the following order: whey ≥ soy ≫ casein; and the following leucine content: whey > casein > soy resulting in leucinemia and hypothetical intracellular leucine concentrations. Therefore, a greater and more rapid rise in blood and, probably, intramuscular leucine concentration triggers a greater rise in MPS. Values are mean ± SE. *MPS* muscle protein synthesis, *FSR* fractional synthetic rate, *IC* intracellular, *[Leucine]* concentration of leucine, * significantly different (*p* < 0.05) vs. casein (one-way analysis of variance), ^*†*^ significantly different (*p* < 0.05) than soy (one-way analysis of variance)

Much of the evidence reviewed above has led to the proposal of the leucine ‘trigger’ hypothesis [22] that revolves around the concept that leucine is the key amino acid that triggers a rise in MPS [18, 45]. As such, proteins that are richer in leucine would be more effective than proteins with lower leucine content [46]. In addition, the rapidity of digestion, and thus the peak leucinemia, would be an important consideration as it would dictate the supply of leucine to trigger MPS. This concept is shown in Fig. 2b and highlights the fact that exercise generally increases the sensitivity to leucine and thus lowers the leucine threshold, whereas aging [46] and inactivity [47] increase the threshold, and the muscle takes on a state of ‘anabolic resistance’ of MPS to hyperleucinemia and hyperaminoacidemia in general. Current evidence would thus lead to a guideline stating that to achieve peak rates of MPS, a high leucine-containing protein that is rapidly digested, leading to rapid leucinemia and hyperaminoacidemia, should be consumed post-exercise.

## High(er) Protein in Weight Loss

A number of studies have compared higher than normally consumed (i.e. ~15–17 % of total dietary energy intake from protein) protein intakes in their effects on weight loss. While there is little doubt that the energy deficit per se will determine weight loss, the focus with weight loss and higher protein diets should be more on what is referred to as the ‘quality’ of the weight loss [48]. The operational definition of weight loss quality is loss of a high ratio of fat to lean tissue, with an emphasis on the loss of visceral fat [49]. Therefore, while general conclusions regarding weight loss in long-term free-living individuals have suggested that weight loss is no different with higher protein intakes [50, 51], short-term trials have shown important differences in the weight lost as fat with muscle ‘spared’ [48]. What is evident is that with respect to weight loss with exercise, higher protein and higher dairy protein in particular provide a protective effect for muscle, even allowing its accrual in certain circumstances [49, 52]. An important observation from an exercise performance standpoint is that in a group of overweight women who consumed a higher protein diet (1.3 g/kg BM/day) during diet and exercise-induced weight loss, they experienced greater gains in strength [49, 53]. However, protein is not able to ablate the loss of skeletal muscle mass completely, especially if the energy deficit is substantial and rapid weight loss occurs, even in exercising athletes [52, 54]. However, when weight loss is more moderate then higher protein intake (1.6 g/kg BM/day) cannot only preserve lean mass but allow performance gains [52]. Unfortunately, without continued supervision, the same athletes who lost fat and gained muscle in the 8-week study period returned to their pre-intervention body composition after 12 months [55].

## Adjunctive Nutritional Strategies to Augment Muscle Protein Synthesis

While it is clear that aminoacidemia following protein ingestion drives the rise in MPS, other nutrients have been added to protein in an attempt to augment its impact on MPS. Carbohydrates have been a primary focus in this area, with the rationale that their energy may serve to reverse an exercise-induced suppression of protein synthesis, either by activation of adenosine monophosphate kinase [56] or through a calcium–calmodulin-dependent mechanism [57]. Alternatively, insulin as a result of carbohydrate ingestion could either promote protein synthesis, suppress proteolysis, or both [58]. However, to date several studies combining protein and carbohydrate have shown no augmentation of protein synthesis when protein is provided in adequate amounts [13, 14, 59]. However, these data do not preclude the hypothesis that carbohydrate is not stimulatory with lower-than-optimal protein doses. In addition, the restoration of muscle glycogen by means of carbohydrate ingestion is also obviously important for athletes and should not be neglected.

Only a few amino acids have been tested in their capacity to augment MPS, but none has proved beneficial in young men. Glutamine (0.3 g/kg BM) was given to young men following 90 min of cycling at 65 % of peak oxygen uptake in addition to carbohydrate and balanced EAA, and there was no difference in post-exercise MPS compared with the placebo trial [60]. The lack of an effect of glutamine on MPS following endurance exercise is at odds with data showing that even endurance exercise is anabolic for mitochondrial and myofibrillar protein synthesis [61]. Congruent with the absence of any benefit of glutamine on MPS after endurance exercise are data from young men performing resistance training who received glutamine throughout 6 weeks of training (0.9 g/kg lean tissue/day) [62]. Glutamine supplementation has been shown to be useful in certain clinical populations, in whom there is a relative lack of intracellular glutamine [63]. However, it is perhaps not overly surprising that glutamine is ineffective in populations who have adequate levels of the amino acid, because it is hard for even a high dose of glutamine to increase intramuscular glutamine [60], and conclusions of recent reviews have been that glutamine appears to be far from useful for athletes [64].

As a precursor for nitric oxide biosynthesis, the amino acid arginine has received some attention for its potential role to promote blood flow and enhance nutrient or hormonal delivery to muscles allowing enhanced anabolism [65–67]. The one study in which MPS has been measured in humans following exercise with arginine supplementation showed no effect of a bolus dose (10 g) of arginine on nitrate or nitrite concentration, femoral artery flow, or MPS [65]. An interesting observation was that growth hormone concentrations were enhanced by arginine supplementation [65] but, similar to other studies [68, 69], the transiently increased growth hormone concentration did not enhance MPS. Other attempts to enhance blood flow after resistance exercise by means of arginine or other nitric oxide-enhancing compounds have proved unsuccessful, at least in healthy young men [66, 67].

## Conclusion

Changes in MPS are variable throughout the day on a meal-to-meal basis, and are augmented immediately and for a prolonged time period after resistive exercise. Endurance exercise also stimulates MPS, but the responses are different to those with resistance exercise, and there is far less clarity on the length of time that they persist. Dietary protein appears to be most effective when consumed after exercise, to take advantage of the ‘receptive state’ of the muscle, for mounting a robust MPS response. This would appear to be a guideline that athletes engaging in resistance and endurance training should follow to allow the synthesis of new proteins specific to their activity, and also to promote adaptive remodeling and repair of any cellular damage. The dose of protein that appears most effective following resistance exercise, and possibly endurance exercise, is approximately 0.25–0.30 g protein/kg BM/meal, at least when consuming isolated proteins. Leucine is a key amino acid in stimulating MPS and its content in, for example, whey protein is probably a primary reason why whey protein is so effective at stimulating MPS as opposed to isolated soy and casein proteins. Therefore, proteins containing a high content of leucine that are digested rapidly are most effectively directed toward MPS; however, ingestion of foods such as milk promote a robust stimulation of MPS and highlight the fact that ‘blends’ of fast and slow proteins are still effective in stimulating MPS. When protein is sufficient, dietary carbohydrate and the ensuing insulinemia does not augment the response of MPS, but carbohydrate is still a practical macronutrient to consume to promote glycogen resynthesis. Neither arginine nor glutamine have been demonstrated to be effective at promoting resistance exercise-induced anabolism in humans and their inclusion in supplements has, on the basis of current evidence, no grounds.
